# A Dairy-Derived Ghrelinergic Hydrolysate Modulates Food Intake In Vivo

**DOI:** 10.3390/ijms19092780

**Published:** 2018-09-15

**Authors:** Ken Howick, Shauna E. Wallace-Fitzsimons, Dalia Kandil, Barbara Chruścicka, Mert Calis, Eoin Murphy, Brian A. Murray, Ayoa Fernandez, Kate M. Barry, Phil M. Kelly, Aoife M. Ryan, John F. Cryan, Brendan T. Griffin, Harriët Schellekens

**Affiliations:** 1 Department of Anatomy and Neuroscience, University College Cork, Cork T12 YT20, Ireland; k.howick@umail.ucc.ie (K.H.); shauna.wallace@umail.ucc.ie (S.E.W.-F.); dkandil@ucc.ie (D.K.); barbara.chruscicka@ucc.ie (B.C.); Mert-c@live.nl (M.C.); j.cryan@ucc.ie (J.F.C.); 2 School of Pharmacy, University College Cork, Cork T12 YT20, Ireland; brendan.griffin@ucc.ie; 3 Food for Health Ireland, University College Cork, Cork T12 YT20, Ireland; 4 Alimentary Pharmabiotic Centre (APC) Microbiome Ireland, University College Cork, Cork T12 YT20, Ireland; 5 Moorepark Food Research Centre, Teagasc, Fermoy, Co. Cork P61 C966, Ireland; Eoin.Murphy@teagasc.ie (E.M.); Brian.Murray@kerry.com (B.A.M.); ayoa.fernandez@teagasc.ie (A.F.); kate.barry@abbott.com (K.M.B.); philk51@hotmail.com (P.M.K.); 6 Department of Food & Nutritional Sciences, University College Cork, Cork T12 YT20, Ireland; A.Ryan@ucc.ie

**Keywords:** ghrelin, ghrelin receptor, bioactive peptides, dairy, food intake, appetite, calcium mobilisation, cachexia, malnutrition

## Abstract

Recent times have seen an increasing move towards harnessing the health-promoting benefits of food and dietary constituents while providing scientific evidence to substantiate their claims. In particular, the potential for bioactive protein hydrolysates and peptides to enhance health in conjunction with conventional pharmaceutical therapy is being investigated. Dairy-derived proteins have been shown to contain bioactive peptide sequences with various purported health benefits, with effects ranging from the digestive system to cardiovascular circulation, the immune system and the central nervous system. Interestingly, the ability of dairy proteins to modulate metabolism and appetite has recently been reported. The ghrelin receptor (GHSR-1a) is a G-protein coupled receptor which plays a key role in the regulation of food intake. Pharmacological manipulation of the growth hormone secretagogue receptor-type 1a (GHSR-1a) receptor has therefore received a lot of attention as a strategy to combat disorders of appetite and body weight, including age-related malnutrition and the progressive muscle wasting syndrome known as cachexia. In this study, a milk protein-derivative is shown to increase GHSR-1a-mediated intracellular calcium signalling in a concentration-dependent manner in vitro. Significant increases in calcium mobilisation were also observed in a cultured neuronal cell line heterologously expressing the GHS-R1a. In addition, both additive and synergistic effects were observed following co-exposure of GHSR-1a to both the hydrolysate and ghrelin. Subsequent in vivo studies monitored standard chow intake in healthy male and female Sprague-Dawley rats after dosing with the casein hydrolysate (CasHyd). Furthermore, the provision of gastro-protected oral delivery to the bioactive in vivo may aid in the progression of in vitro efficacy to in vivo functionality. In summary, this study reports a ghrelin-stimulating bioactive peptide mixture (CasHyd) with potent effects in vitro. It also provides novel and valuable translational data supporting the potential role of CasHyd as an appetite-enhancing bioactive. Further mechanistic studies are required in order to confirm efficacy as a ghrelinergic bioactive in susceptible population groups.

## 1. Introduction

The potential health benefits of the bioactive fragments which exist within the matrix of many food and dietary components have long been known [1]. Milk is one of the richest sources of bioactive fragments and there is a growing body of evidence that these can have positive effects on appetite and metabolism [2,3,4,5]. Many of these bioactives are proven to have various health benefits, spanning the digestive, endocrine, cardiovascular, immune and nervous systems [6,7]. Identification and isolation of these bioactives, as well as elucidating their pharmacodynamic parameters are necessary to realise functional applications [8]. The utilisation of dairy-derived bioactives in appetite-related disorders is now becoming increasingly apparent [1,2]. The ability of a bioactive to enhance satiety and decrease food intake in vivo has been shown [4]. Conversely, recent work has also shown a whey protein isolate to reduce the expression of satiating genes in the hypothalamus and to increase food intake in rodents [5]. However, more translational studies are required to provide insights into the merits and mechanisms of milk-derived bioactives to treat appetite-related disorders.

The endogenous hormone ghrelin, a 28 amino acid peptide is one of the key factors involved in food intake regulation [9]. The ghrelin receptor growth hormone secretagogue receptor-type 1a (GHSR-1a) has thus been a therapeutic target for disorders of appetite [10,11]. In particular, the focus has been on individuals with poor appetite secondary to co-morbid conditions such as cardiovascular disease, respiratory disease and cancer, who can suffer an advanced form of ‘wasting syndrome’ known as cachexia [12]. Though ghrelin and synthetic ghrelin ligands have shown robust effects on food intake in humans for the treatment of cancer-related cachexia, the initiation of pharmaceutical therapy is restricted to these patients and would not be routine in mildly reduced appetites, such as those seen in ageing populations [13,14,15]. Nevertheless, a ubiquitous decline in ghrelin levels with age is a major contributory factor to appetite reductions and weight loss [16]. Poor nutritional status in elderly patients is a complicating factor for chronic diseases and results in prolonged hospital stays, lessened independent living and poorer response to treatment, leading to an overall greater burden on global health infrastructures and poorer clinical outcomes [17]. Age-related appetite loss hence represents an important, unmet, clinical need. Given the lack of suitable pharmacotherapy and the growing role of nutraceutical science, we suggest the potential role of a bioactive ghrelin agonist to help delay the onset of a cachectic state due to comorbid illnesses.

Bioactives that augment the ghrelin system have previously yielded anecdotal evidence of increased appetite, which has since been substantiated by animal and human studies. Rikkunshito (RKT), a long-standing traditional Japanese herb has the ability to function as a ghrelin agonist [18]. RKT has been shown to reduce weight loss and increase food intake in mouse models of wasting syndrome [19,20], while a retrospective analysis of cancer patients showed increased median survival time in patients receiving concomitant RKT with their treatment [18]. Chin-shin oolong tea, a popular tea in Taiwan, was perceived to induce hunger, and subsequently was shown to increase food intake in rats [21]. In vitro, an isolate from emoghrelin heshouwu, a Chinese traditional medicine, was shown to activate the GHSR-1a and stimulate growth hormone (GH) secretion in vitro, supporting a claim for its perceived therapeutic efficacy as an anti-ageing supplement [22]. Furthermore, an extract from the herbal medicine *Harpagophytum procumbens* was shown to act on the GHSR-1a and modulate appetite in an in vivo mouse model [23].

There is an impetus to provide dietary-incorporated, scientifically validated interventions for poor appetite at an early point, rather than initiating late-stage pharmaceutical therapy which is often expensive, ineffective and not without side-effects. The proactive use of nutraceutical therapy as a preventative or complementary approach to traditional pharmacotherapy has been recently discussed [24,25]. Milk is one of the primary sources of bioactive fragments for nutraceuticals, many of which have purported health benefits in relation to appetite and metabolism. However, further bioactive identification, enrichment and incorporation into appropriate delivery systems is required [26]. Our screening platform revealed the dairy hydrolysate (CasHyd), a mixture of peptides produced by the enzymatic cleavage of casein, which potently activates the GHSR-1a in vitro. We demonstrate additive and synergistic effects of the hydrolysate with ghrelin. We also investigate the potential of this bioactive peptide mixture to function as an appetite stimulant in vivo under different modes of administration. This study thus represents an interesting, novel translational investigation of a dairy-derived appetite-stimulating bioactive targeting the GHSR-1a with potential for inclusion as a functional food ingredient in population groups with poor appetite who may be at risk of developing malnutrition and cachexia.

## 2. Results

### 2.1. Activity of CasHyd on Ghrelin-Receptor Overexpressing-Cell Line

**Figure 1:**
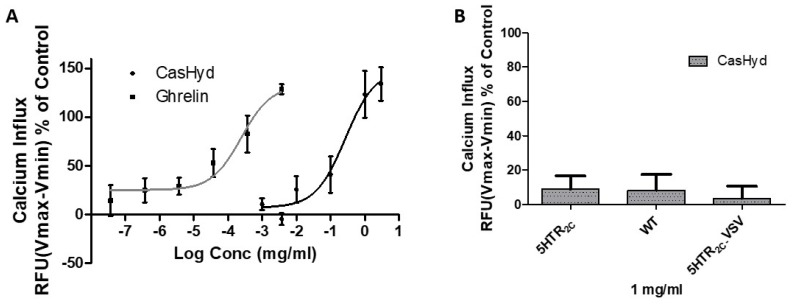
(**A**) Concentration response curve of casein-derived hydrolysate. Concentration response curve for the casein-derived hydrolysate, CasHyd, measured in growth hormone secretagogue receptor-type 1a (GHSR-1a) overexpressed in HEK293A cells; (**B**) Activity of CasHyd in wild-type (HEK293A-WT) cells, 5HT_2C_ receptor (HEK293A-5HTR_2C_) and a fully edited form of 5HTR_2C_ (HEK293A-5HTR_2C_ -VSV) expressing cells. Intracellular Ca^2+^ increase is depicted as a percentage of maximal Ca^2+^ influx in relative fluorescence units (RFU) as elicited by control (3.3% fetal bovine serum; FBS). The parent casein protein elicited negligible effects on all cell lines. Graphs represent mean ± SEM (standard error of the mean) of at least three independent experiments performed in triplicate.

The activity of the casein-derived hydrolysate, CasHyd, on the GHSR-1a was analysed using an intracellular Ca^2+^ mobilisation assay, as a measure of downstream GHSR-1a signalling activation [27], in HEK293A cells stably expressing the GHSR-1a tagged with an enhanced green fluorescent protein (GHSR-1a-EGFP) (Figure 1).

CasHyd stimulated calcium mobilisation in cells expressing GHSR-1a in a concentration-dependent manner, with the EC_50_ = 0.27 mg/mL and efficacy (E_max_) reaching 148.9%. The potency of CasHyd was 1000-fold lower than that for the endogenous receptor ligand, ghrelin (EC_50_ = 0.25 µg/mL, E_max_ = 132.5%) (Table 1). Considering CasHyd is a mixture of different peptides not all of which are likely to elicit bioactivity, the activation found here on the GHSR-1a indicates promising ability to modulate the receptor. Efficacy of CasHyd was expressed as a percentage of the maximum response obtained by the positive control (3.3% fetal bovine serum, FBS, E_max_ = 100%). Interestingly, no concentration-dependent Ca^2+^ mobilisation was observed in wild-type HEK293A cells (HEK293A-WT) not expressing the GHSR-1a. Furthermore, no activity was observed in 5HT_2C_ (HEK293A-5HTR_2C_) receptor-expressing cell line, nor in the edited from of the 5HTR_2C_, (HEK293A-5HTR_2C_-VSV), compared to treatment with control (FBS), which gives maximal intracellular Ca^2+^ mobilisation in all tested cell lines. Food intake and adiposity are altered in vivo when the 5-HT_2C_ receptor RNA is fully edited, suggesting a potential role for 5-HT_2C_ editing in eating disorders [28]. Together, these results show, to our knowledge for the first time, the promising potential of a casein hydrolysate, CasHyd, to modulate the GHSR-1a.

### 2.2. Calcium Imaging on Ghrelin-Receptor Overexpressing-Cell Line

**Figure 2:**
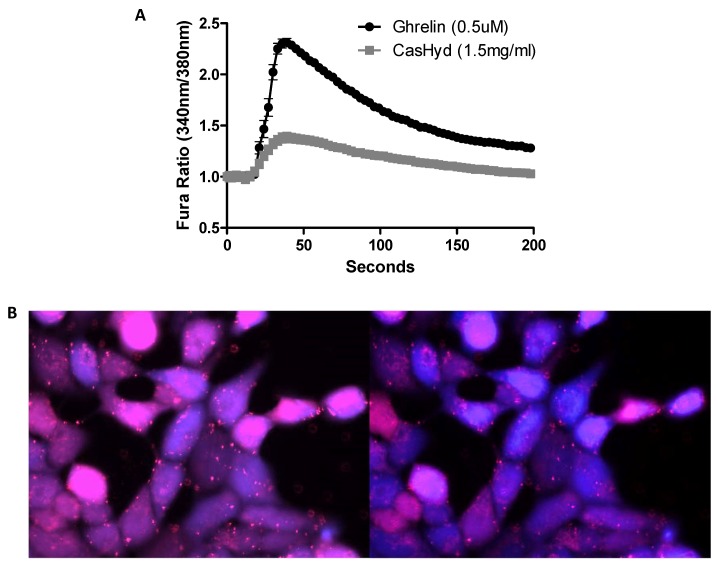
Specific activation of heterologously expressed GHSR-1a in HEK293A cell line. (**A**) Calcium imaging of human embryonic kidney (HEK) cells heterologously expressing the GHS-R1a. Cells were seeded for 48 h into wells at a density of 2.0 × 10^5^ cells/mL, and loaded for 1 h with the UV-excitable fluorescent calcium indicator, Fura-2AM, and the 340 nm/380 nm ratio was recorded after addition of 500 nM ghrelin or 1.5 mg/mL CasHyd. (**B**) Calcium influx is evident through the shift in fluorescence from pink to blue (40× magnification). Traces represent the average of three independent experiments, error bars are indicative of SEM.

Next, the GHS-R1a mediated calcium response of the HEK-GHSR-1a cells to ligand exposure was investigated using calcium imaging. Following addition of 500 nM ghrelin, an increased fluorescence peak is observed indicating calcium influx due to treatment. This calcium influx is also observed upon the acute addition of CasHyd (1.5 mg/mL), but to a lesser extent than was seen upon ghrelin addition (Figure 2A). Calcium influx is evident through the shift in fluorescence from pink to blue (Figure 2B), indicating calcium release from intracellular stores resulting in an excitation shift from 340 nm to 380 nm. This further corroborates the calcium mobilisation results obtained.

### 2.3. HPLC Characterisation of CasHyd

**Figure 3:**
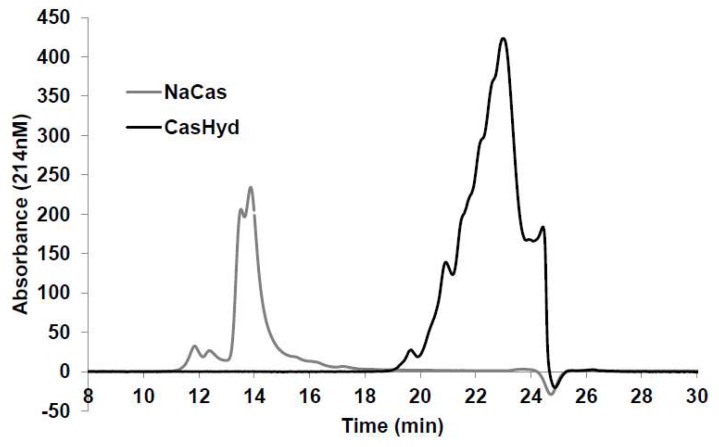
Size exclusion HPLC for CasHyd fraction compared with parent casein protein. Molecular weight distribution of CasHyd and parent protein, sodium caseinate (NaCas), expressed as absorbance over time on HPLC chromatogram.

Size exclusion chromatography carried out on the whole unhydrolysed protein, sodium caseinate (NaCas) versus the hydrolysate, CasHyd, shows no overlap in the molecular weight distribution after enzymatic hydrolysis (Figure 3). The unhydrolysed parent protein, NaCas, showed 85.9% of total proteins to be >25 kDa molecular weight, whereas 86.0% of CasHyd is below 1 kDa in size (Table 2). This shows the extent of hydrolysis which takes place, yielding a mixture of vastly different peptide fractions to the parent casein protein. In addition, the high level of hydrolysis of CasHyd, yielding a majority of peptides <1 kDa, is likely to contribute to the observed bioactivity of GHSR-1a modulation.

### 2.4. GHSR-1a Activation by CasHyd in Neuronal Cells In Vitro

**Figure 4:**
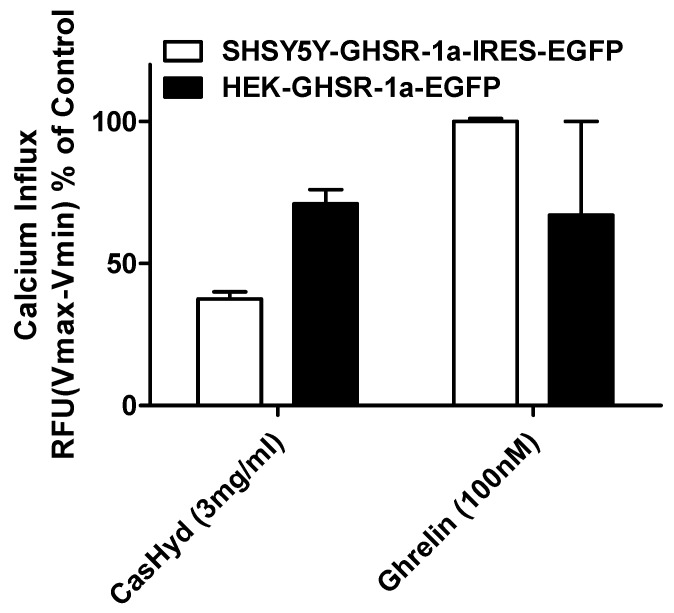
Specific activation of GHS-R1a receptor in vitro. Both CasHyd (3 mg/mL) and ghrelin (100 nM) elicited a GHS-R1a-eGFP (enhanced green fluorescent protein) mediated calcium influx in both HEK cells stably expressing GHSR1a-eGFP, and the neuronal-like SHSY-5Y cell line generated to express GHSR1a-eGFP using lentiviral vectors. Graph represents the mean ± SEM of a representative experiment. Each concentration was carried out in triplicate. Intracellular calcium increase was normalised to maximal calcium increase as elicited by control (100 nM ghrelin).

Next, the activity of CasHyd was assessed in the neuroblastoma cell line, SHSY-5Y, engineered to overexpress the GHS-R1a as a native receptor using lentiviral vectors. A calcium mobilisation response following exposure to the endogenous ligand, ghrelin, as well as the dairy-derived hydrolysate, CasHyd, was observed in both engineered cell lines (Figure 4).

### 2.5. Additive and Synergistic Effects of the Dairy-Derived Hydrolysate on Ghrelin-Mediated GHSR-1a Activation

**Figure 5:**
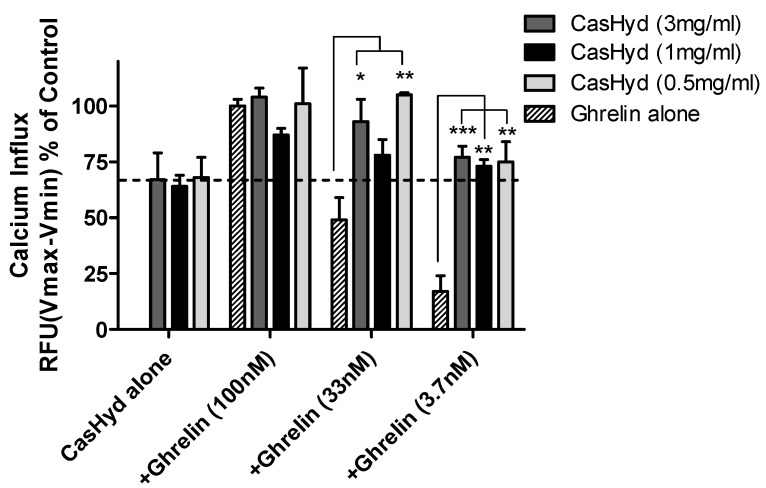
Additive GHS-R1a activation following co-treatment of ghrelin and CasHyd. Additive calcium mobilisation is observed following co-treatment of HEK cells stably expressing the GHS-R1a-eGFP with 33 nM of ghrelin and CasHyd. Graph represents the mean ± SEM of a representative experiment of three independent experiments. Each concentration reported was carried out in triplicate. Intracellular calcium increase was normalised to maximal calcium increase as elicited by control (100 nM ghrelin). Statistically significant differences were calculated using a one-way analysis of variance (ANOVA) followed by the Bonferroni post-hoc test for multiple comparisons and are depicted as *** *p* < 0.001, ** *p* < 0.01 and * *p* < 0.05.

**Figure 6:**
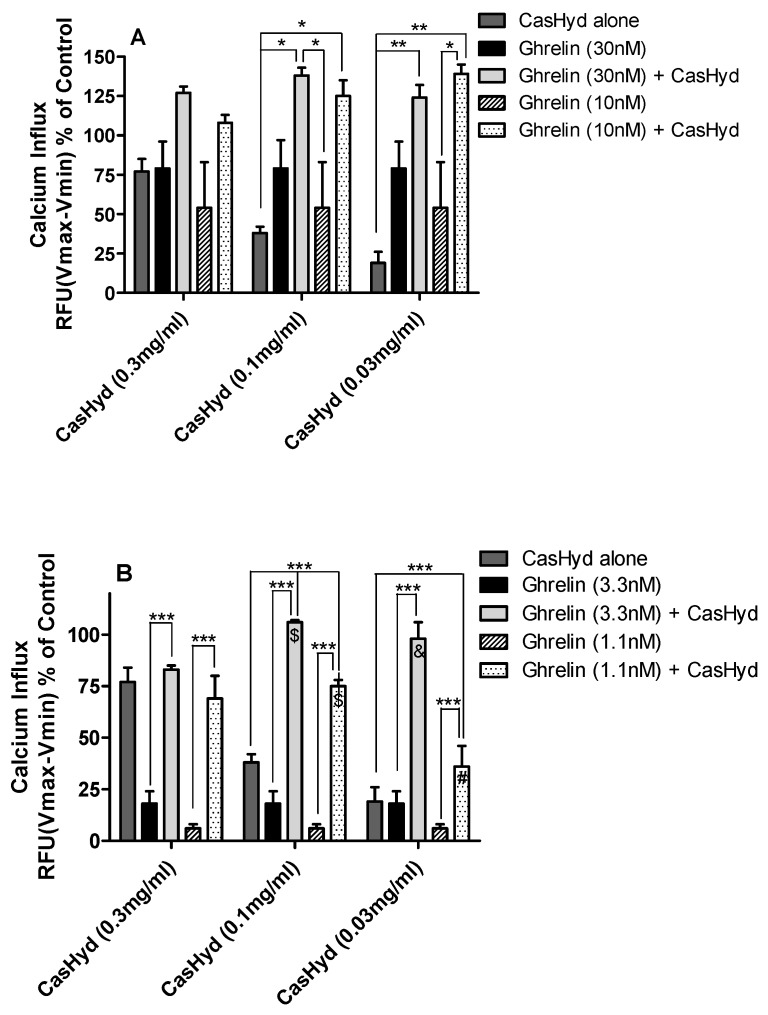
Additive and synergistic effects of GHS-R1a activation following co-treatment of ghrelin and CasHyd. Additive and synergistic effects are observed following co-treatment of HEK cells stably expressing the GHS-R1a with hydrolysate and 30 nM or 10 nM ghrelin (**A**), or hydrolysate and 3.3 nM or 1.1 nM ghrelin (**B**). Intracellular calcium increase was normalised to the maximum calcium increase as elicited by control (100 nM ghrelin). Graphs represent the mean ± SEM of a representative experiment of three independent experiments, with each concentration point performed in triplicate. Statistically significant differences of combination treatment indicating an additive effect were calculated using a one-way analysis of variance (ANOVA) followed by the Bonferroni post-hoc test for multiple comparisons and are depicted as *** *p* < 0.001, ** *p* <0.01 and * *p* <0.05. Statistically significant differences of a synergistic effect are depicted as; $ *p* < 0.001, & *p* < 0.01 and # *p* < 0.05.

Potential additive or synergistic effects between ghrelin and the dairy-derived hydrolysate on GHS-R1a activation were investigated. Cells stably expressing the GHSR1a were exposed to different concentrations of ghrelin (100–3.7 nM) and CasHyd (3–0.5 mg/mL). Increases in intracellular calcium could be observed following all concentrations of hydrolysate and a dose dependent calcium influx for ghrelin (Figure 5). However, no synergistic effects were observed. A small additive effect was observed for cells treated with a suboptimal concentration of ghrelin (33 nM) and all three concentrations of CasHyd but this did not reach statistical significance. However, when analysing the effect on calcium mobilisation using lower concentrations of CasHyd (0.1 and 0.03 mg/mL), clear additive effects could be observed with 30 and 10 nM ghrelin (Figure 6A). In addition, additive calcium mobilisation was observed following co-treatment of 0.3 mg/mL CasHyd and 3.3 or 1.1 nM ghrelin (Figure 6B). Moreover, synergistic effects were observed when cells were co-treated with the two lowest concentrations of CasHyd (0.1 or 0.03 mg/mL) and ghrelin (3.3 and 1.1 nM).

### 2.6. Cumulative Food Intake Studies after Intraperitoneal Injection of Peptide Solution

**Figure 7:**
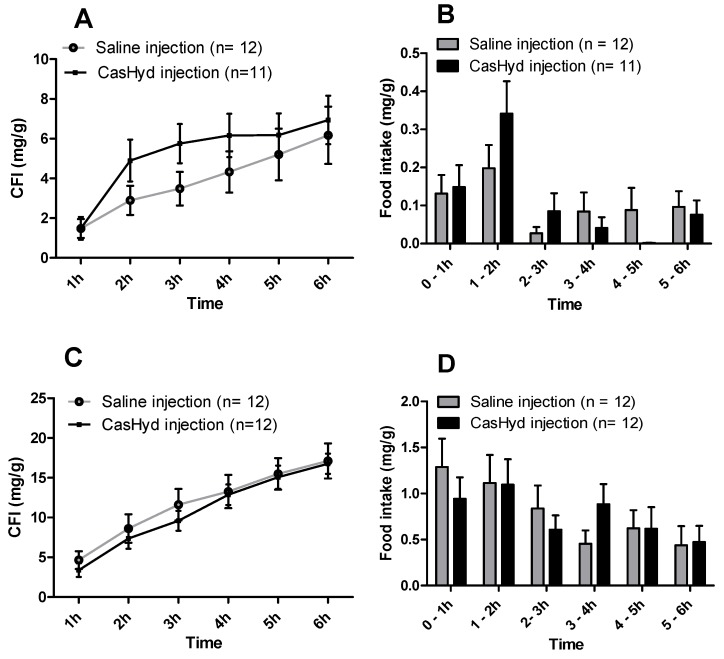
Cumulative food intake following intraperitoneal administration of dairy-derived peptide hydrolysate. Cumulative food intake (CFI) (regular chow) intake in male (**A**) and female (**C**) Sprague-Dawley rats was determined following intraperitoneal injection with 50 mg·kg^−1^ body weight of CasHyd over 6 h. The food intake per time bin is also illustrated for males (**B**) and females (**D**). Data presented as mean ± SEM.

In food intake studies following an intraperitoneal injection (IP) of CasHyd 50·mg kg^−1^ in 0.9% saline, or control, there were no significant differences noted in the amount of food consumed between groups, normalised to body weight (Figure 7). Examination of individual time bins yielded no overall differences at any timepoint. Hence, we conclude that CasHyd is not effective as an appetite stimulant with this mode of delivery.

### 2.7. Cumulative Food Intake Studies after Oral Administration of Peptide Solution

**Figure 8:**
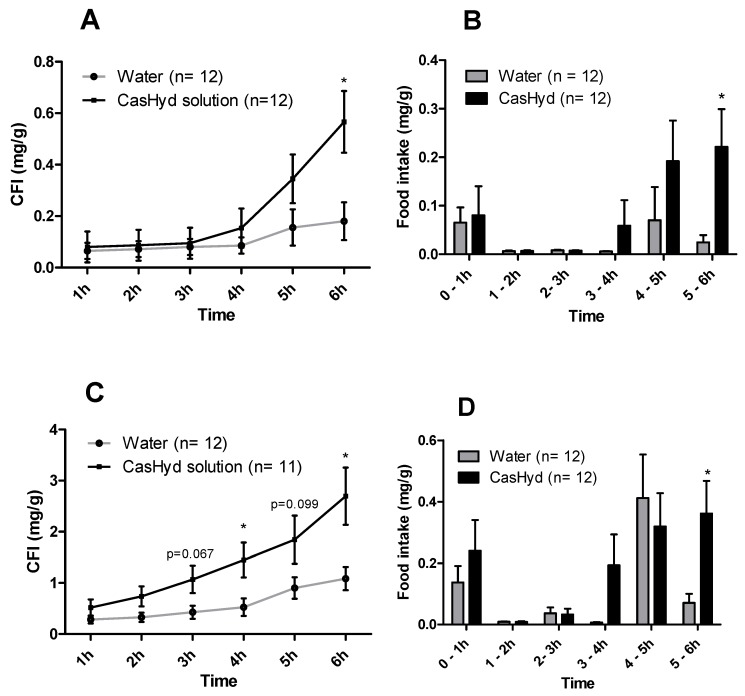
Cumulative food intake following oral administration of unencapsulated dairy protein-derived hydrolysates. Food (regular chow) intake in male (**A**) and female (**C**) Sprague-Dawley rats was determined following oral gavage with 50mg kg^−1^ body weight of CasHyd over 6 h. Cumulative food intake (CFI) was determined at regular intervals after oral gavage. The food intake per time bin is also illustrated (**B**,**D**). Graphs represent the mean ± SEM. Statistical significance was determined using repeated measures ANOVA and estimation of parameters for food intake. Pairwise comparisons were carried out using Tukey’s post-hoc test, while independent samples t-tests were used for each individual time bin; statistical significance is depicted as * *p* < 0.05.

In cumulative food intake (CFI) studies following an oral gavage of a 50 mg·kg^−1^ dose of peptide solution, there were significant increases noted in the amount of food consumed relative to control in both males and females, normalised to body weight (males, *p* = 0.013, females *p* = 0.021; Huyn-Feldt sphericity correction) (Figure 8). Pairwise comparisons also revealed trends at 3 h and 5 h post dose, and a significant increase at 4 h compared to control for the female cohort. The most significant change in food intake was in the 5–6 h time bin for both males and females (Figure 8B,D). The GHSR-1a is located on vagal nerve terminals in the gastrointestinal tract, throughout the small and large intestine [29], potentially explaining the increased efficacy of the oral route versus IP via potential local GHSR-1a stimulation.

### 2.8. pH Susceptibility of CasHyd

**Figure 9:**
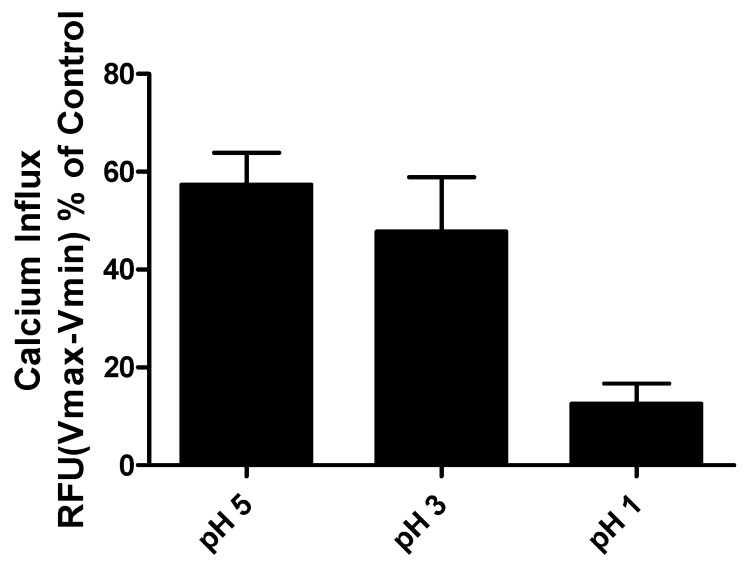
GHSR-1a agonist, CasHyd, displays pH dependent activity. Reduction in hydrolysate-mediated GHSR-1a activation following exposure to acidic pH confirms the need for an oral delivery mechanism. The graph represents three independent experiments carried out in at least triplicate (control = CasHyd not exposed to acidic pH, RFU = relative fluorescence units).

CasHyd was exposed to increasing levels of acidity for a period representative of the minimum amount of time required for gastric emptying (30 min [30]). A pH-dependent loss in peptide activity is observed for CasHyd, confirming the need to develop a gastro-resistant formulation to minimise exposure to gastric acid before progression to further in vivo efficacy studies (Figure 9).

### 2.9. Delivery System (Pellet) Characterisation Work

**Figure 10:**
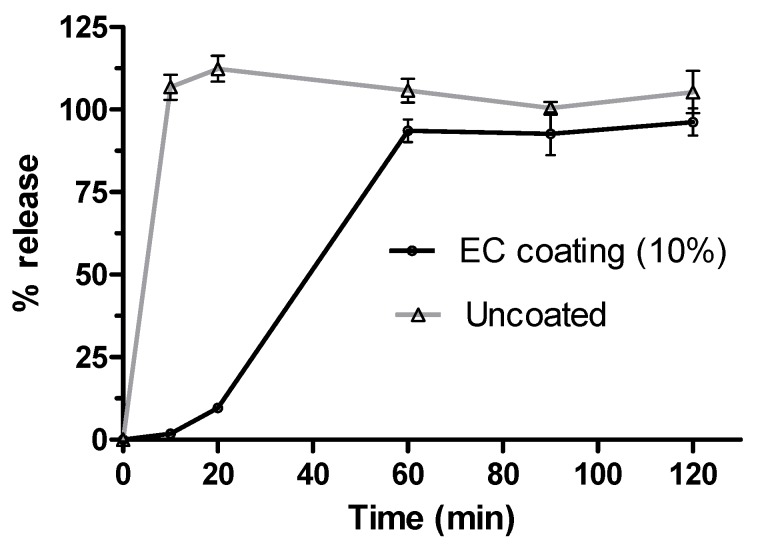
Dissolution study of gastro-protected CasHyd pellets. USP type 1 (basket) dissolution studies (50 rpm, 37.5 °C) showed gastro-protected release in simulated conditions (simulated gastric fluid sine pepsin, SGFsp; pH 1.2).

Since CasHyd is susceptible to acidic pH, the peptide mixture was incorporated into a gastro-protected delivery system (coated pellets) in order to minimise exposure to the stomach in vivo. Simulated release profile assessment of CasHyd from the formulation was carried out in vitro in order to assess whether the coating applied to the pellets was able to delay release. USP Type 1 (basket) dissolution studies were carried out in gastric conditions (simulated gastric fluid, SGFsp, pH 1.2) in order to confirm a delayed release of peptide from the pellets. Pellets displayed a delayed release of peptide load over 60 min, confirming the functionality of the delivery system (Figure 10).

### 2.10. GHSR-1a Activity of Peptide Post-Encapsulation

**Figure 11:**
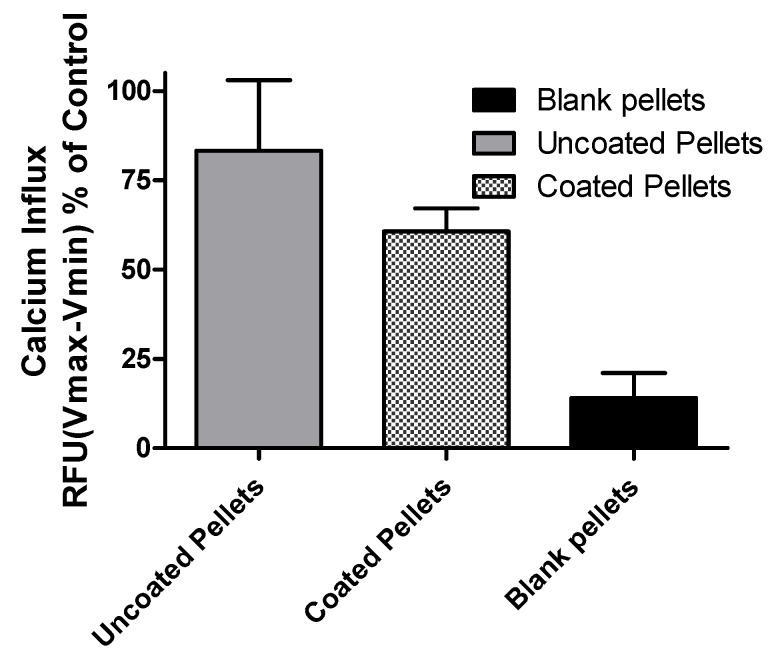
CasHyd retains bioactive functionality after encapsulation. Activity of CasHyd after encapsulation was determined relative to activity of non-encapsulated CasHyd in GHSR-1a- expressing cells. Activity was quantified as being 75% for uncoated pellets and 60% for coated pellets (representative of three independent experiments carried out in triplicate).

Due to the likely fragile nature of the peptide hydrolysate [26], we quantified the impact of the encapsulation processing conditions on bioactivity. Activity of CasHyd in the encapsulated pellets was determined relative to activity of non-encapsulated CasHyd peptide in the GHSR-1a overexpressing cells, as described above. Activity was quantified as being 75% for uncoated pellets and 60% for coated pellets (Figure 11).

### 2.11. Cumulative Food Intake Studies after Oral Administration of Peptide Encapsulated in Gastro-Protective Pellets

**Figure 12:**
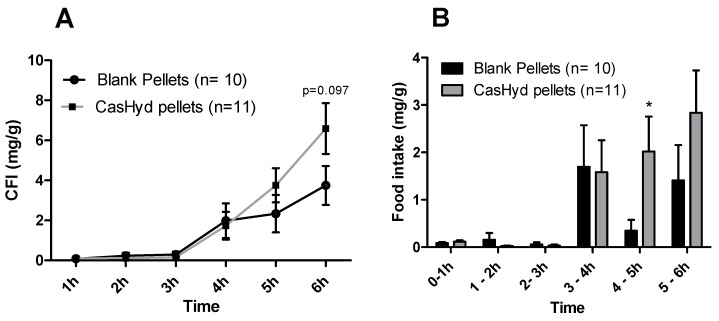

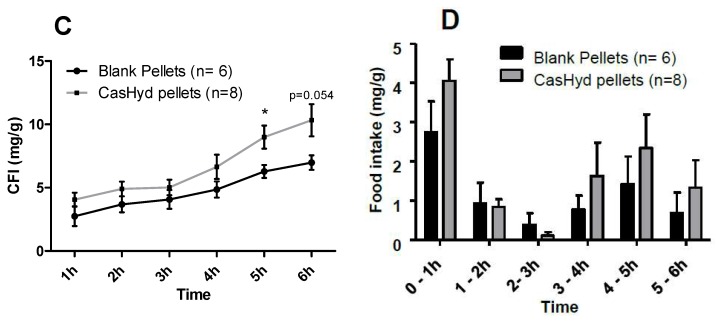
Cumulative food intake following oral administration of gastro-protective pellets containing casein-derived hydrolysate. Food (regular chow) intake in male (**top**) and female (**bottom**) Sprague-Dawley rats was determined following oral gavage with 35 mg kg^-1^ dose of encapsulated CasHyd over 6 h. Cumulative food intake (CFI) was determined at regular intervals, beginning 4 h after oral gavage of the coated pellets containing CasHyd, or an equivalent weight of blank pellets (**A**,**C**) The food intake per time bin is also illustrated (**B**,**D**). Graphs represent the mean ± SEM. Statistical significance was determined using repeated measures ANOVA and estimation of parameters for food intake. Pairwise comparisons were carried out using Tukey’s post-hoc test, while an independent samples *t*-test was used for each individual time bin; statistical significance is depicted as * *p* < 0.05.

In food intake studies following an oral gavage of casein hydrolysate encapsulated in a coated pellet formulation vs. a blank pellet formulation, there were no overall significant increases noted in the amount of food intake for males or females; however, a trend towards an overall increase is noted at the 6 h timepoint for both (Figure 12). A significant increase in amount of food consumed was observed in the 4–5 h time bin for the male cohort also. However, the orexigenic effect seen after oral dosing of the unencapsulated peptide is not confirmed here.

## 3. Discussion

The endogenous hormone ghrelin, and its receptor, GHSR-1a, are of vital importance in maintaining energy homeostasis and appetite regulation [11,31]. Numerous studies report orexigenic effects after administration of the hormone [32,33]. Furthermore, natural analogues of ghrelin have provided anecdotal and, more recently, experimental evidence of a positive effect on appetite and energy balance in susceptible population groups [18]. Hydrolysates of milk proteins, both casein and whey, are increasingly recognised for their bioactive components which may bestow therapeutic benefits on appetite [1,2,5,34]. A casein-derived bioactive fraction with specific serotonin-2C receptor (5-HT_2C_) agonist activity eliciting satiating properties in a rodent model has been described [4]. In this study, we demonstrated that a casein hydrolysate displayed intrinsic GHSR-1a agonist activity which translated to an effect on increasing food intake in vivo in rats.

The dairy hydrolysate, CasHyd, dose-dependently and specifically increased intracellular Ca^2+^ in HEK293A cells heterologously expressing the GHSR-1a. We have previously reported ghrelin agonistic effects of a whey-based protein derivative in the same in vitro system [26]. The CasHyd described here displays superior potency (0.27 mg/mL) compared to the whey derived fraction; however it is considerably less than the endogenous GHSR-1a ligand (0.25 µg/mL), ghrelin (Figure 1). This is likely reflective of the fact that CasHyd is a mixture of peptides, only some, or one, of which may be active on GHSR-1a. Interestingly, the in vitro activity has negligible effects on WT, 5HTR_2C_ or the fully edited form of 5HTR_2C_. The activity of CasHyd is also shown to be both additive and synergistic to native ghrelin in vitro (Figure 5 and Figure 6). Furthermore, the activity of CasHyd was assessed in the neuroblastoma cell line, SHSY-5Y, engineered to overexpress the GHS-R1a receptor as a native receptor using lentiviral vectors. A calcium mobilisation response following exposure to the endogenous ligand ghrelin as well as the dairy-derived hydrolysate was observed in both this cell line, and that of HEK293A (Figure 4). This reinforces the GHS-R1a activating potential of the hydrolysate. This provides promising evidence to further examine CasHyd activity on GHSR-1a in a physiologically relevant environment using primary cultured neuronal cells. HPLC-SEC contrasted the size differences of the parent casein protein and CasHyd, confirming the efficacy of the hydrolysation process (Figure 3), while HPLC was also used to confirm the reproducibility of the hydrolysation process (Figure S2). The high presence of low molecular weight peptide sequences (<1 kDa, Table 2) is critical to the bioactivity reported in these assays.

This is the first instance in which a casein-derived peptide has been shown to have GHSR-1a agonist properties in vitro. Furthermore, an increase in food intake in vivo is reported after dosing with CasHyd. This may be due to a translation of GHSR-1a stimulation from in vitro to in vivo, although further studies must be done to confirm this. Nevertheless, we show that CasHyd displays evidence of enhancing food intake in healthy Sprague-Dawley (SD) rats. Male and female rats treated orally using a solution of CasHyd at a dose of 50 mg/kg showed a three-fold increase in food intake over the six-hour experiment (Figure 8); however, this is tempered by a relatively low quantity of food consumed overall. Time bins illustrate a significant elevation in both groups in the 5–6 h timepoint, potentially indicative of a prolonged/delayed systemic effect. Interestingly, following IP injection of CasHyd (50 mg/kg dose), neither male nor female rats displayed a significant increase in food intake relative to control (Figure 7). The apparent success of oral delivery of the bioactive peptide relative to injection may be reflective of the distribution of the GHSR-1a in vivo, which is heavily expressed in the gastrointestinal tract and involved in neuronal signalling to appetite centres in the brain [26].

Despite the apparent increase in food intake after oral gavage of CasHyd, in vitro assays confirm that acidic pH, comparable to that experienced in the gastric conditions, is detrimental to CasHyd bioactivity (Figure 9). The ability of bioactive peptides to elicit a beneficial effect in vivo is hence likely to be highly dependent on the use of a gastro-protective delivery system [35,36]. This is in line with recent literature highlighting the role of drug-delivery research strategies for bioactive materials [36,37]. Therefore, we sought to develop a gastro-protective formulation to minimise acid-mediated degradation of the casein fraction and enhance delivery to the small intestine. A coated pellet formulation was established, utilising extrusion-spheronisation for pelletisation, followed by coating with an ethylcellulose-based polymer using fluidised bed technology. CasHyd encapsulated in a coated oral delivery vehicle (pellets) showed a trend towards an increase in food intake in female rats (*p* = 0.054), and male rats (*p* = 0.097) at the 6-h timepoint, although overall no significant differences are noted. Furthermore, the absolute amount of food consumed in the experimental period is higher after dosing with pellets (Figure 12) compared with CasHyd solution (Figure 8), which may be reflective of the bulk volume of pellets; it may be that dosing pellets which slowly disintegrate in the intestine creates a paradoxical increase in food intake, thereby confounding any comparisons to orally dosed solutions. Furthermore, the orally dosed pellets impact on the timing of the release of the bioactive which may in itself lead to different effects; i.e., the immediate availability of the peptide in the stomach vs. the gradual release from slowly dissolving pellets.

Overall, although food intake results showed high variability, these initial proof-of-concept studies represent promising results. The increase in food intake after oral gavage of CasHyd is tempered by efforts to substantiate the claim as an appetite stimulant by incorporating it into a gastro-protected vehicle. These efforts did not find any such increase, in either food intake or in the additional blood biomarkers reported in the supplementary data (Figure 12 and Figure S1). Further discussion on the study limitations is therefore merited, specifically in relation to the suitability of the experimental setup for assessing food intake, and peptide release characteristics from the delivery system.

Firstly, although the food intake model described has been reported in previous studies involving a bioactive peptide, food intake in rodents is inherently variable, and susceptible to change by a multitude of factors. Inter-experimental variability is evident in the differing absolute amount of food consumed between studies. Healthy, normophagic rats were used in this study making it difficult to observe any increases in food intake, given that metabolic drive would generate a healthy appetite by default. All experiments were also carried out in the light phase, when rodents normally would be asleep—circadian fluctuations may serve here as a confounder to assessing true appetite. Furthermore, the dosing procedure exerted a degree of restraint stress upon the animals, while there is a risk of minor local injury to the oesophagus in gavaged rats which is also likely to impact on food intake. Secondly, the bioactive hydrolysate itself is likely to be highly fragile in vivo, due to low gastric pH (discussed above), as well as intestinal peptidases. Variability in results may well be a consequence of systemic breakdown. Thirdly, in the case of pelletised CasHyd, the delivery system design incorporated the peptide into a gastro-protected pellet which exerted a degree of processing stress on the peptide, resulting in a loss of ~40% bioactivity. The bulk effect of solid pellets also seems to have imparted a default increase in food intake in both males and females compared to oral solution. While this formulation was useful as proof of concept, process optimisation is required to minimise activity losses, reduce bulk volume and tailor the release profile further in vivo.

Despite the above described caveats to this study, hitherto, a lot of evidence substantiating nutraceutical and bioactive health claims comes from in vitro bioinformatics, with many lacking tangible in vivo evidence of effect [1,38]. Therefore, evidence is needed to further support the claim of dairy-derived bioactives for appetite modulation. Our casein-derived bioactive peptide, CasHyd, shows promising novel results translating a specific in vitro bioactivity with high potency, to a promising biofunctional effect on food intake in vivo, suggesting overall success of this proof of concept study. Given a more suitable platform for assessment of food intake, and/or an optimised oral delivery mechanism to improve stability during formulation, a considerable potential to increase food intake in vivo by targeting intestinal GHSR-1a exists.

The area of bioactives for appetite modulation is of growing commercial interest and has the potential to address an unmet clinical need by providing an evidence-based, dietary incorporated, early intervention for conditions of undereating. CasHyd is a GHSR-1a agonist which represents a novel nutraceutical approach to increasing appetite in susceptible populations. However, further work must be done in order to fully elucidate its clinical merit, while technology to retain and enhance activity in vivo is also required.

## 4. Materials and Methods

### 4.1. Materials

Dairy-derived peptide hydrolysate (CasHyd) was provided by Food for Health Ireland (see Section 2.2). Disposable plastic flexible gavage tubes were purchased from Instech Laboratories (Instech Laboratories, Inc. Plymouth Meeting, PA, USA). Standard chow (2018S Teklad Global 18% Protein Rodent Diet) was procured from Harlan, UK Ltd. (Bicester, UK). For encapsulation of the bioactive, an aqueous pseudo-latex of EC (Surelease^®^ Type B NF) was sourced from Colorcon Corp., Indianapolis, Indiana. Microcrystalline cellulose (MCC, Avicel^®^ PH-101 NF Ph. Eur.) was purchased from FMC Corp., Little Island, Cork, Ireland and pharmaceutical grade ethanol 96% (*v*/*v*) from Carbon Chemicals Group Ltd., Ringaskiddy, Cork, Ireland. For the Ca^2+^ mobilisation assays, fetal bovine serum (3.3%) was obtained from Sigma-Aldrich, Arklow, Wicklow, F7524, Ireland. The assay buffer was composed of 1x Hanks balanced salt solution, HBSS, Gibco™ 14065049 (Thermo Fisher Scientific™, Waltham, MA, USA), containing 20 mM HEPES (Sigma-Aldrich, Arklow, Wicklow, Ireland). The endogenous agonist, ghrelin (rat), was supplied by Tocris Bioscience, Avonmouth, Bristol, UK (Cat. No. 1465).

### 4.2. Generation of CasHyd

Sodium caseinate (NaCas, Kerry Group Plc, Listowel, Ireland) was suspended at 10% (*w*/*w*) on a protein basis in water and dispersed under agitation 50 °C for 1 h using an in-line mixer (total batch size—1000 L). Protein hydrolysis was carried through addition of food grade enzyme for a duration of 3 h at 50 °C. The pH of hydrolysis was maintained at a constant pH for the duration of hydrolysis by addition of a hydroxide base (Microbio, Fermoy, Ireland). The enzyme was then inactivated by heat treatment through a plate and frame heat exchanger (Unison Engineering Services Ltd., Limerick, Ireland). Large molecular weight material and aggregates were removed from the hydrolysate through membrane separation or clarification steps. The clarified material was then ultrafiltered at 50 °C using 1 kDa spiral wound organic membranes (Synder Filtration, Vacaville, CA, USA) operating under a transmembrane pressure of 2 bar. A diafiltration step using reverse osmosis was utilised in order to increase recovery of small peptides in the permeate. The permeate fraction (CasHyd) was dried in a single stage spray dryer (Anhydro F1 Lab Dryer, Copenhagen, Denmark).

### 4.3. Ca^2+^ Mobilisation Assay for Peptide GHSR-1a Activity

GHSR-1a mediated changes in intracellular Ca^2+^ were recorded on a High-Throughput Cellular Screening System (Molecular Devices Corporation, Sunnyvale, San Jose, CA, USA). Ca^2+^ mobilisation assays were performed according to a protocol modified from a previously described method [39]. Stably transfected human embryonic kidney (HEK293A) cells overexpressing GHSR-1a were seeded in sterile 96-well microtiter plates with black-walled and clear-bottomed wells (3904, Costar, Fisher Scientific, Dublin, Ireland) at a density of 2.5 × 10^4^ cells per well. Cells were then kept at 37 °C in a humidified atmosphere containing 5% CO_2_ overnight. Twenty-four hours before the experiment, the medi was replaced with serum-free media (1% non-essential amino acids). On the day of the assays, cells were allowed to incubate with 80 µL of 1× Ca5 dye dye (R8186, Molecular Devices) in assay buffer (1× Hanks balanced salt solution—HBSS, supplemented with 20 mM HEPES buffer). CasHyd was dissolved in assay buffer (1× HBSS supplemented with 20 mM HEPES buffer). Addition of the dissolved compounds (25 µL/well) was performed automatically. Fluorescent readings were taken for 120 s at excitation wavelength of 485 nm and emission wavelength of 525 nm. The percentage increase in cytosolic Ca^2+^ was deduced from the difference between basal and maximal fluorescence and illustrated as a percentage of maximum response (reading from 3.3% fetal bovine serum; FBS). Background fluorescence from assay buffer alone was subtracted from all readings. The endogenous agonist ghrelin (1465; Tocris) was also used as a positive control of Ca^2+^ influx. Data were analysed using GraphPad Prism software (PRISM 5.0; GraphPAD Software Inc., San Diego, CA, USA). Nonlinear regression analysis with variable slope was used to generate sigmoidal concentration-response graphs.

### 4.4. Calcium Imaging

Calcium imaging took place for HEK-GHSR-1a cells seeded on a 12-well plate at 2.0 × 10^5^ cells/mL two days before the assay according to a previously described method [39]. The day before the assay, media was swapped to serum-free. For the assay procedure, all media was removed from cells which were then washed using phosphate-buffered saline and incubated for 1 h at 37 °C with 7 µM Fura 2-AM (F1221, Biosciences) in assay buffer. Upon calcium release, the fluorescent excitation maximum of the Fura-2 indicator undergoes a blue shift from 363 nm (Ca^2+^-free) to 335 nm (Ca^2+^-saturated), while the fluorescence emission maximum remains unchanged at 510 nm. Upon excitation at 340 nm and 380 nm respectively, the ratio of the fluorescent intensity emissions at these excitations is correlated to the levels of intracellular calcium. Subsequently, media was replaced with assay buffer without Fura 2-AM. Cells were viewed and a field was selected under brightfield illumination (Olympus BX50WI, Tokyo, Japan). Standard digital epifluorescence system (Cell R, Olympus) was used to measure changes in intracellular calcium (Ca^2+^). Light at 340 and 380 nm was generated using a Xenon/Mercury arc burner (MT20 illumination system, cell R, Olympus), illuminating the cells and stimulating fura 2 fluorescence. Hydrolysates or the endogenous GHS-R1a receptor agonist ghrelin (SP-GHRL-1, Innovagen, Lund, Sweden) were added and the excitation spectra at 380 nm (Ca^2+^-free) and 340 nm (Ca^2+^-saturated) with fixed emission at 510 nm were recorded.

### 4.5. HPLC Characterisation of CasHyd

CasHyd and its parent protein (NaCas) were analysed using size exclusion (SE) high-performance liquid chromatography using a TSK G2000SWXL 7.8 × 300 mm column (Tosoph Corporation, Tokyo, Japan). Analysis was carried out at isocratic conditions for 40 min; the mobile phase was 30% *v*/*v* and 0.1% *v*/*v* TFA in deionised water. Flow rate through the column was 0.5 mL/min. The total injection volume was 20 mL. Absorbance of the eluate was measured at 214 nm. The following molecular weight standards were used for calibration purposes: Tyr-Glu (310 Da); Leu-Trp-Mel-Arg (605 Da); bacitracin (1.4 kDa); aprotinin (6.5 kDa); α-lactalbumin (14.2 kDa); and, bovine serum albumin (66 kDa).

Different batches of CasHyd were analysed using both reverse-phase (RP) high-performance liquid chromatography. The HPLC equipment consisted of an Agilent 1200 series chromatograph (Agilent Technologies, Santa Clara, CA, USA) equipped with an auto-sampler, quaternary pump and a multiple wavelength detector. RP-HPLC was carried out using a Zorbax 300SB-C18 column (Agilent Technologies, USA). Two mobile phases were used A: 0.1% *v*/*v* tri-fluoro acetic acid (TFA) in deionised water and B: 0.1% *v*/*v* in acetonitrile. A linear solvent gradient was applied, ramping from 3 to 60% B over 20 mins, 60 to 95% B over the following 4 mins, holding and 95% B for 2 min before a final decrease from 95 to 3% B over 2 min. Solvent flow rate was 1 mL/min. Solutions (2000 ppm; 15 mL) were loaded onto the column which was equilibrated at 35 °C. The column eluate was monitored at 214 nm.

### 4.6. Cell Culture, In Vitro Transfection and Lentiviral Transduction

Hek293A and SHSY5Y cells were maintained in culture in high glucose Dulbecco’s modified Eagle’s medium (Invitrogen, Carlsbad, CA, USA) enriched with heat-inactivated fetal bovine serum and non-essential amino acids at 10% and 1% respectively, in an atmosphere of 95% air and 5% CO_2_ at 37 °C. Hek293A cells were transfected using lipofectamine LTX plus reagent (Invitrogen) with a GHS-R1a-EGFP construct (EX-X0963-M03, Genecopeia, Rockville, MD, USA) according to manufacturer’s instructions. Cells stably expressing the GHS-R1a receptor with the C-terminal-EGFP fusion protein, were selectively cultured with geneticin (G418, Merck, Kenilworth, NJ, USA.) as a selection antibiotic. Those cells showing the greatest fluorescence were sorted using flow assisted cell sorting (FACS). In addition, SHSY5Y cells were transduced to express the GHS-R1a receptor using a 3rd generation packaging, gene delivery and viral vector production system developed by Naldini and colleagues [40,41,42,43]. HIV-based lentivector (LV) particles expressing the GHS-R1a from a spleen focus-forming virus (SFFV) promoter in conjunction with an EmGFP sequence expressed as a separate protein after an internal ribosome entry site (IRES) were generated. Briefly, the GHS-R1a sequence was cloned into a HIV-based, replication deficient, lentiviral expression plasmid, pHR-SIN-BX-IRES-EmGFP (kind gift of Adrian Thrasher, Institute of Child Health, London, United Kingdom), modified to exclude the shRNA U6 promoter. The GHS-R1a gene was amplified, gel isolated using the Qiagen Gel Extraction Kit (#28706) and ligated into the lentiviral vector using BamHI and XhoI restriction sites, generating pHR-GHS-R1a-IRES-EmGFP. Lentivector (LV) GHS-R1a expressing particles, pseudotyped with the vesicular stomatitis virus G (VSV-G) were produced using 293T-17 cell following transient cotransfection of the cloned expression constructs, pHR-GHS-R1a-IRES-EmGFP, the packaging construct, pCMV∆R8.91 and the envelope construct, pMD.G –VSVG. SHSY-5Y cells were transduced with the GHS-R1a expressing lentiviral vectors diluted in transduction media, consisting of DMEM with 2% heat-inactivated FBS, 1% NEAA and an additional 8μg/mL polybrene^®^ (Sigma; #H9268). Fluorescence was monitored using flow cytometry as indicator of receptor expression.

### 4.7. Cumulative Food Intake Studies

Male and female Sprague-Dawley (SD) rats were purchased from Envigo, UK. Rats were 7 to 8 weeks-old upon arrival in the animal unit. Rats were group-housed at 4 per cage in an environment controlled for light-dark cycle (12-h light; lights on at 7:00 a.m.), temperature (21 ± 1 °C) and humidity (55 ± 10%). Water and standard lab chow (2018S Teklad Global 18% Protein Rodent Diet, Envigo, Huntingdon, UK) were available ad libitum. All experiments were in full accordance with the European Community Council directive (86/609/EEC) and approved by the Animal Experimentation Ethics Committee of University College Cork (B100/3774). Animals were habituated to experimental conditions for a week prior to experiments taking place. On experimental day, animals were administered their respective treatment at the onset of the light phase and then placed in individual cages for duration of food intake monitoring. Food intake was then recorded by weighing the chow at defined intervals. For the gastro-protected pellets, animals were food restricted for a period of 4 h before a pre-weighed quantity of chow was added to the cages. The dosing system for pellets consisted of a flexible PVC gavage tube which was filled with a pre-weighed quantity of blank or active pellets. After insertion of the dosing tube a guidewire was used to administer the dose of pellets directly into the stomach.

### 4.8. Pellet Preparation by Extrusion-Spheronisation

Requisite quantities of CasHyd and MCC were combined in a ratio of 33:67 and manually blended for 1 min. A Kenwood planetary mixer (KM005, Kenwood Ltd., Hampshire, UK) was then used to further dry blend the mixture for 5 min at a minimum agitation setting. The dry powder blend was gradually wetted by adding deionised H_2_O, under constant agitation by the planetary mixer. The granulation end-point was achieved upon addition of a cumulative amount of deionised H_2_O equivalent to 45% (*w*/*w*) of the dry powder blend. The granulate was immediately extruded at an extrusion speed of 17–19 rpm using a sieve extruder (Caleva^®^ Extruder 20, Caleva Process Solutions, Sturminster Newton, UK). Screen thickness and aperture diameter were both 1 mm. The extrudate was then placed into a Caleva^®^ Spheroniser 250 for 90 s at 1500 rpm (Caleva Process Solutions, Sturminster Newton, Dorset, UK). Pellets were collected and dried using high flow air in a microfluid bed system (Vector Corp., Marion, IA, USA) at 40 °C for 20 min before coating took place.

### 4.9. Pellet Film Coating

Film coating was performed in a laboratory scale microfluid bed system in bottom-spray mode. Nozzle air was set to 16–17 psi and airflow was 310–335 L/min. Coating solution, an 11% (*w*/*w*) aqueous pseudo-latex of EC (Surelease^®^ Type B), was fed at a constant rate (1.0 g/min). Prior to coating, the Surelease polymer was allowed to homogenise for 30 min under constant agitation. Uncoated pellets were charged to the coating vessel and pre-heated for 10 min with an inlet air temperature of 80 °C, such that sufficient drying could be obtained of the coating polymer. This was achieved at an outlet air temperature of ~50 °C. The amount of coating polymer required for film coating was calculated as a theoretical percentage weight gain based on the weight of the uncoated pellets at the start of coating. The microfluid bed coating system was constantly monitored to ensure that appropriate air flow and drying was maintained in the coating chamber.

### 4.10. pH Susceptibility Tests

CasHyd was dissolved in deionised H_2_O and acidified with HCl to the requisite pH (pH 1, 3, 5 and untreated), using a pH enomenal^®^ 1000 L pH meter and electrode. Acidified CasHyd solutions were incubated for 30 min under gentle agitation. 50 µL of each sample was removed and added to 950 µL of Ca^2+^ assay buffer and pH checked to confirm that acidity was neutralised before samples were added to cells.

### 4.11. In Vitro Dissolution Studies:

Dissolution testing (USP Type 1) was performed, using a basket-type dissolution apparatus (DISTEK, Inc., Model 2100C, North Brunswick, NJ, USA). Simulated gastric fluid sine pepsin (SGFsp) (pH 1.2, 500 mLs) was used as dissolution media. Dissolution bath temperature was kept at 37 °C and 50 rpm agitation speed. Sampling was conducted at various timepoints. After each sample an equal volume of dissolution medium was added to the dissolution vessel to maintain volume at 500 mL.

### 4.12. Peptide Quantification Assay

The bicinchoninic acid (BCA) assay was performed using a BCA assay kit (Thermo Fisher Scientific™ Pierce™ BCA Protein Assay, Catalog Number 23225) according to a well-established method. A 2 mg/mL stock solution of CasHyd in SGFsp was used to prepare a series of dilutions for preparation of a standard curve (2, 1, 0.5, 0.25, 0.125, and 0.0625 mg/mL, respectively). 25 µL of each sample obtained during dissolution testing, and standards were plated on a 96-well plate. After the dissolution experiment was completed, remaining pellets were removed, crushed, and quantified as above in order to confirm all peptide was released from the formulation. Working reagent was prepared by mixing BCA assay Reagent A with BCA assay Reagent B in a ratio of 50:1. The working reagent (200 µL) was then transferred to each well. The plates were then covered and incubated at 37 °C for 30 min. Spectrophotometric analysis was performed at 562 nm and the quantity of peptide in each sample was quantified from standard curves and expressed as a percentage of total peptide in the pellets.

### 4.13. Data Analysis

Data were analysed and graphs generated using GraphPad Prism software and Microsoft Excel software. For in vitro cell screening and dissolution work, means were the result of at least three independent experiments performed in triplicate. Calcium mobilisation assays report the standard error of the mean (SEM) while the dissolution result reports standard deviation (SD). For in vivo food intake, measurements between groups were analysed using a one-way, repeated measures ANOVA followed by estimation of parameters. If data were non-spherical as determined by Mauchly’s test for sphericity, a Huynh-Feldt correction was applied for data analysis. Graphs are expressed as mean ± SEM. Statistical significance was indicated as follows: * indicates *p* < 0.05; ** indicates *p* < 0.01 & *** indicates *p* < 0.001.

## 5. Conclusions

This work describes a dairy-derived peptide with potent activity on the GHSR-1a in vitro. In vivo preclinical studies with this bioactive peptide show its potential to act as an appetite stimulant after oral administration. CFI was increased three-fold after 6 h in male and female SD rats after a single oral dose. However, while activity of CasHyd was eliminated following exposure to gastric pH, administration of CasHyd in a gastro-protected pellet formulation only showed a trend towards increased food intake in both males and females. Variable results may be reflective of the suboptimal release of peptide coupled with loss of bioactivity in vivo, and/or potential lack of suitability of the model to assess subtle appetitive changes in a normophagic rat cohort. Overall, high in vitro efficacy on the GHSR-1a has translated to evidence of an effect on food intake in vivo. Therefore, we consider this study a valuable contribution to the growing body of evidence for nutraceuticals and nutraceutical encapsulation platforms, which will serve as a useful reference for further investigations in preclinical models of age-related malnutrition or cachexia.

## Figures and Tables

**Table 1:** Activity of CasHyd and Ghrelin on GHSR-1a overexpressing HEK293A cells.

Compound	EC_50_	E_max_^1^
Ghrelin	0.25 µg/mL (74 nM)	132.5%
CasHyd	0.27 mg/mL	148.9%

^1^ Intracellular Ca^2+^ increase reported as a percentage of maximal Ca^2+^ influx in relative fluorescence unit (RFU) as elicited by control (3.3% FBS).

**Table 2:** Molecular weight distribution of NaCas and CasHyd.

Molecular Weight	Retention Time (min)	NaCas (% Area)	CasHyd (% Area)
>25 kDa	<14.66	85.9	0.0
25–10 kDa	14.66–17.26	13.1	0.0
10–5 kDa	17.26–19	1.0	0.0
5–1 kDa	19–21.35	0.0	14.0
<1 kDa	>21.35	0.0	86.0

## Supplementary Materials

Supplementary materials can be found at http://www.mdpi.com/1422-0067/19/9/2780/s1.

## Abbreviations

GHSR-1a	Growth Hormone Secretagogue Receptor-Type 1a
CasHyd	Casein Hydrolysate
RKT	Rikkunshito
HEK	Human Embryonic Kidney
RFU	Relative Fluorescence Unit
EGFP	Enhanced Green Fluorescent Protein
FBS	Fetal Bovine Serum
CFI	Cumulative Food Intake
SGF	Simulated Gastric Fluid
