# Supplementary material for: A Dairy-Derived Ghrelinergic Hydrolysate Modulates Food Intake In Vivo
Source: Int J Mol Sci. 2018 Sep 15;19(9):2780. doi: 10.3390/ijms19092780 (PMC6165545; doi:10.3390/ijms19092780)
Supplement: Supplementary file 1 [file ijms-19-02780-s001.pdf]

Blood Biomarker Analysis

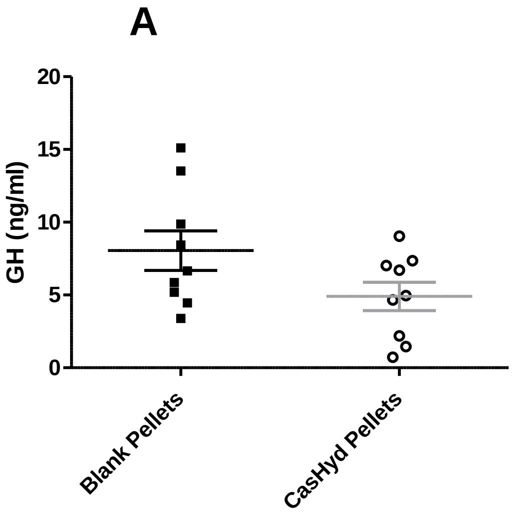

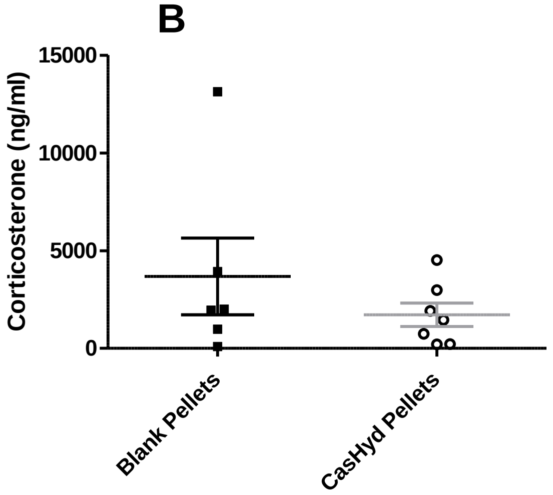

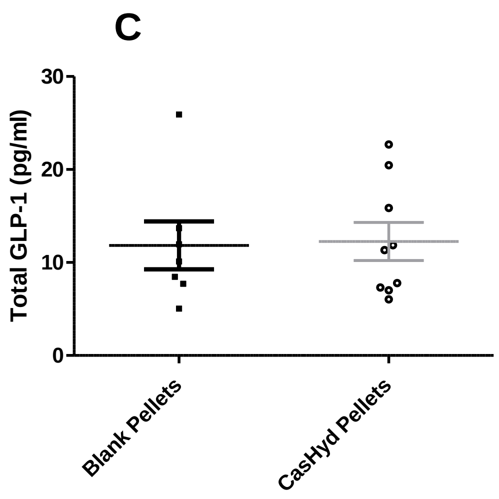

Figure S1. Enzyme linked immunosorbent assay for growth hormone **(A)**, corticosterone **(B)**, and total GLP-1 **(C):** 4 hours post-dosing with CasHyd pellets animals were euthanised and trunk blood collected for analysis. Growth hormone (GH), total glucagon-like peptide (GLP-1) and corticosterone (cort). No significant differences were detected between the treatment and control animals 4 hours post-dosing with either CasHyd-loaded pellets or blank pellets.

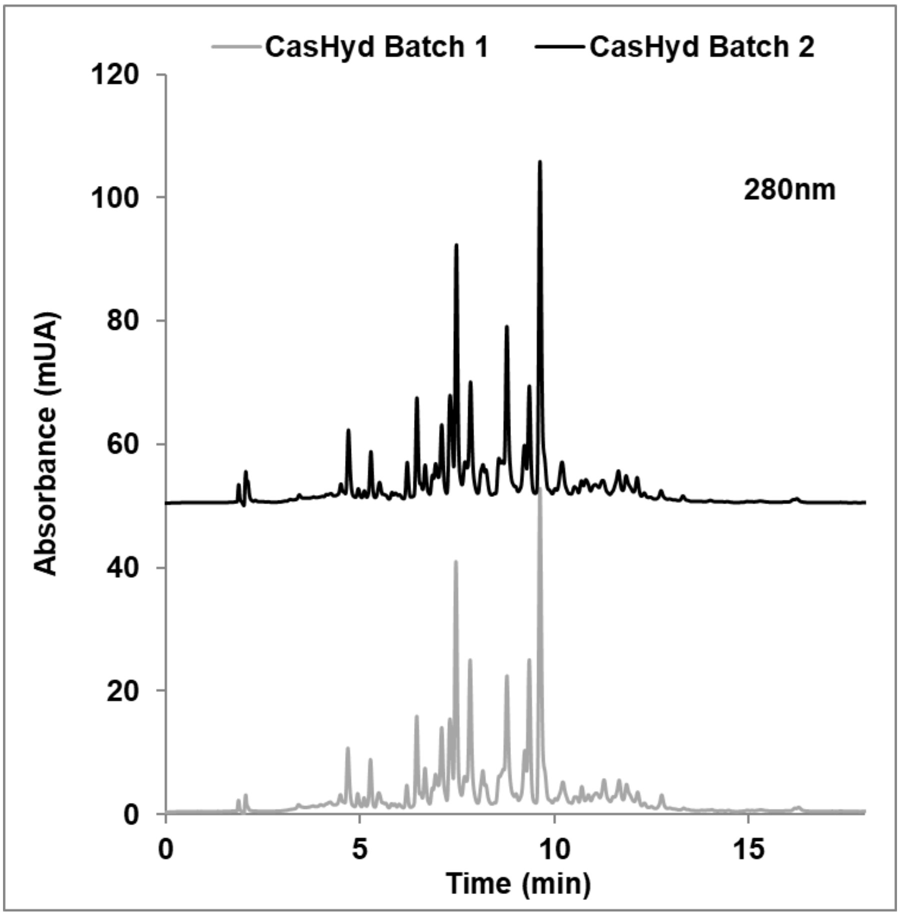

Figure S2. Reverse-phase HPLC analysis of two different batches of CasHyd. HPLC chromatograms of two different batches of CasHyd illustrating almost identical profiles, indicating the reproducibility of the hydrolysation process. Chromatograms expressed as absorbance at 214nm over time.
